# Improved DNA extraction from marine bones: A 15-year retrospective evaluation using an adapted Dabney extraction method

**DOI:** 10.1007/s00414-026-03923-w

**Published:** 2026-07-17

**Authors:** Nina Schwemer, Sophie Fertyk, Jan Euteneuer, Johanna Preuß-Wössner, Maria Seidel

**Affiliations:** 1 Institute of Legal Medicine, University Hospital Schleswig-Holstein, Kiel, Schleswig-Holstein Germany; 2 Institute of Legal Medicine, University Hospital Essen, Nord-Rhein-Westfalen, Germany

**Keywords:** Marine bones, Degraded DNA, Ancient DNA, Silica membrane

## Abstract

**Background:**

Extracting DNA for the purpose of identification from bone material, especially for old samples or from extreme environmental conditions, has always been a challenging task in forensic genetics. At our forensic institute with two locations between the Baltic Sea and the North Sea, found marine bones are regularly submitted for evaluation and analyzed using molecular biology methods.

**Methods:**

An improvement to commercially available bead-based methods routinely used for the bone investigations and often yielding poor or no DNA, was therefore aimed for. In the present study, an overview of all marine bones analyzed from the years 2009–2024 was conducted and samples – if possible – reanalyzed with a newly introduced adapted extraction method according to Dabney.

**Results:**

A total of 82 bone finds have been examined over the last 15 years, 18 of which (22%) could eventually be re-examined using the adapted Dabney method. Morphologically, 61% of the bone finds were long tubular bones, followed by skulls and mandibular bones with both 13%. Compared to the North Sea bones, the Baltic Sea bones were better preserved on the outside, but showed increased adhesions of algae and barnacles. A comparison with the previously obtained molecular analysis results showed for the majority of the reanalyzed bones significantly higher DNA concentrations up to a 98-fold increase, enabling DNA profiling with an overall better quality (41% complete profiles compared to 12%).

**Conclusion:**

The reanalysis demonstrated that the adapted Dabney method extracts DNA from marine bones more effectively, and thus offers a superior alternative to a commonly used forensic extraction method.

**Supplementary Information:**

The online version contains supplementary material available at https://doi.org/10.1007/s00414-026-03923-w.

## Introduction

**Fig. 1:**
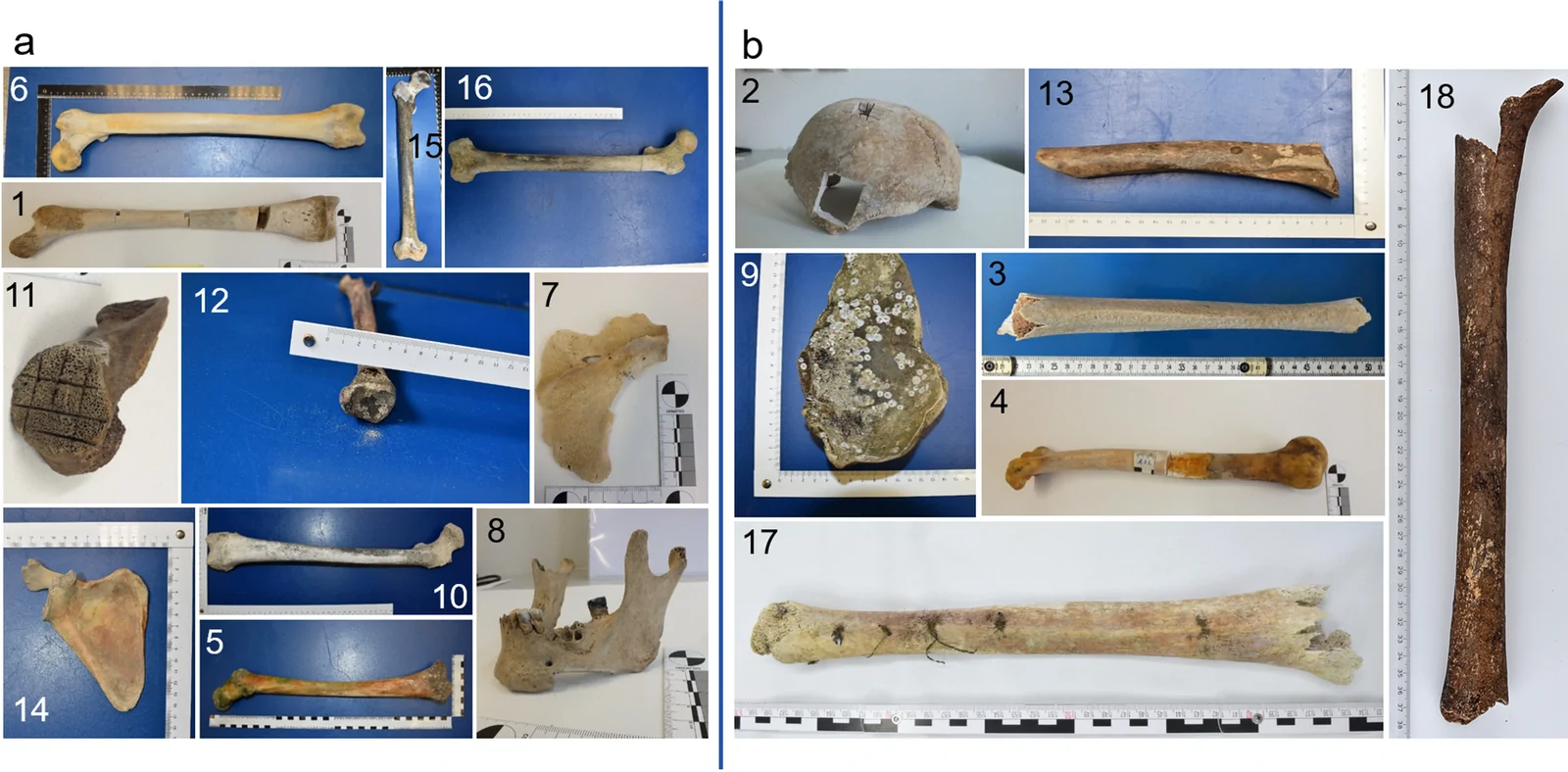
Comparison of the beach finds from the North Sea (**a**) and the Baltic Sea (**b**). Numbering was done chronologically, for a description see Table 1

The geographical location of our institute’s area of responsibility (Schleswig Holstein) between the Baltic and North Seas leads to continuous assignments of marine bone examinations and the question, whether a genetic STR profile can be created for identification purposes. The extraction of DNA from bone or dental material is challenging due to the increased technical effort and small amounts of usually degraded DNA. Therefore, the extraction efficiency is crucial for the success of subsequent investigations. In contrast to recent bones found on or inside the ground, the formerly in-house used bead-based extraction method has rarely been able to obtain sufficient amounts of DNA. In a validation study on (marine) bones, a more effective extraction method based on silica membranes was established: This “Dabney” method has proven to be efficient in numerous aDNA and additionally some forensic laboratories [1–3]. However, a systematic forensic evaluation of the Dabney method for sea water exposed bones with various post-mortem intervals has not been conducted so far. The present study provides a (retrospective) reanalysis of all available and already molecularly analyzed marine bones from recent years (2009–2024) to ascertain whether it is possible to enhance the quantity and quality of extracted DNA from marine bones. The bones from the North Sea generally appeared to be more affected by the prevailing environmental conditions (e.g. higher salinity of the water and tides), as the periosteum was often ablated and the underlying cancellous bone was visible. The bones from the Baltic Sea, on the other hand, showed more frequent adhesions of algae or barnacles (Fig. 1). Where possible, the results of the bead-based method (Bone DNA Extraction Kit from Promega) were compared to the Dabney method (High Pure Viral Nucleic Acid Kit from Roche adapted from Dabney and Meyer [1]), while acknowledging the limitation that for most specimens different sample sites were analyzed and the examinations took place at different times. We aim for a both realistic and comprehensive evaluation of the usability of the Dabney method for the elevated requirements of marine bone analysis. At the same time, an overview of the number and type of in-house investigated beach finds was compiled.

## Materials and methods

### Sample collection and selection

All data and samples were collected retrospectively from the years 2009 to 2024 from the two Institutes of Legal Medicine Kiel and Lübeck. Initially, a comprehensive examination of all archived files was conducted and reports on marine bones selected. In case that a molecular biological examination of the bones had been performed, bones (if still available) were reanalyzed using the Dabney extraction method. The bone discovery sites (Supplement Table 1) were distributed across the entire coasts of Schleswig-Holstein. In total, 30 marine bones from the North Sea and 52 marine bones from the Baltic Sea were reported.

**Table 1:** Comparison of the results of the Dabney and bead-based method of various reanalyzed beach bones. n.a.: no analysis performed; N/A: no analysis possible; * Samples for which no quantification of the DNA concentration was carried out using the bead-based extraction method. S.: small fragment; L.: large fragment

Sample	Bones	Type of samples	Results Dabney [pg/µL]	Results Bead [pg/µL]	Called loci (Dabney/Bead)
1	Femur	New sample site and bone powder	S. 24.45 L. 3.85	S. 12.00 L. 3.10	16 / 16
2	Cranium	New sample site and bone powder	S. 14.95 L. 2.65	n. a. *	16 / 0
3	Tibia	Bone slices from the freezer	S. 4.95 L. 0.40	S. 0.3 L. 0.2	15 / 0
4	Humerus	New sample site and bone powder	S. 2.95 L. 0.20	S. 0.80 L. 0.00	12 / 12
5	Femur	Old bone powder from the freezer	S. 23.40 L. 0.80	S. 0.65 L. 0.20	16 / 13
6	Femur	Bone pieces from the freezer	contaminated	contaminated	contaminated
7	Scapula	New sample site and bone powder	S. 0.00 L. 0.00	n. a. *	N/A / 0
8	Mandibula	New sample site and bone powder	S. 27.25 L. 3.00	n. a. *	16 / n. a.
9	Part of a Cranium	New sample site and bone powder	S. 8.50 L. 0.45	S. 1.60 L. 0.10	16 / 15
10	Femur	New sample site and bone powder	S. 9.80 L. 0.80	S. 0.10 L. 0.00	15 / 3
11	Presumably Femur	New sample site and bone powder	S. 0.00 L. 0.00	S. 0.00 L. 0.00	N/A / 0
12	Tibia	New sample site and bone powder	S. 1.40 L. 0.10	S. 0.00 L. 0.00	5 / 4
13	Femur	New sample site and bone powder	S. 0.00 L. 0.00	S. 0.00 L. 0.00	N/A / 0
14	Scapula	New sample site and bone powder	S. 1.20 L. 0.00	S. 0.00 L. 0.00	3 / k. a.
15	Femur	New sample site and bone powder	S. 19.90 L. 1.25	S. 0.25 L. 0.10	14 / 14
16	Femur	New sample site and bone powder	S. 2.20 L. 0.10	S. 0.70 L. 0.20	11 / inconclusive
17	Tibia	New sample site and bone powder	S. 266.60 L. 18.85	S. 3.10 L. 2.00	16 / 4
18	Femur	New sample site and bone powder	S. 7.05 L. 0.6	S. 7.24 L. 0.00	16 / 16

### Bone preparation

A separate laboratory is maintained solely for the processing of bone samples, which is strictly separated from the routine pre-PCR and post-PCR laboratories. Prior to sample preparation, all surfaces were decontaminated with sodium hypochlorite bleach solution at 0.3%. All instruments were decontaminated before and after use.

If necessary, the bones were cleaned of coarse soiling using a toothbrush. Afterwards, bones were cut into corn kernel-sized pieces using a Dremel rotary tool with a cutting blade. The bone pieces were then soaked in 2.5% diluted sodium hypochlorite solution for 10 min, washed in HPLC grade water for 10 min and dried overnight (min. 16 h, max. 36 h) inside a drying cabinet at 37 °C. The dried bone pieces were grinded in a bone mill (Tube Mill 100 control IKA^®^) using disposable grinding cups. The samples were grinded twice for 1 min at 25,000 rpm. From the resulting bone powder, 100 mg was used for extraction. All bone samples were processed in duplicates. In contrast to the first examination, the selection of the sample site on each bone was based on the working instructions provided by the International Commission on Missing Persons (ICMP) with a catalogue of the most compact bone areas with the best-preserved DNA of the individual bone parts [4], which was published after the study by Hines et al. [5]. As a result, for most of the specimen different sample sites were examined in order to increase the chances of successful extraction.

### DNA-extraction procedures

All steps of DNA extraction were performed with disposable gloves, masks, hairnets and overalls. All members of the institute involved in handling the materials voluntarily provided DNA samples to monitor contamination; however, none was observed. Negative controls were included in all extraction, quantification and PCR reactions. A positive control (bone pieces) was used for the inhibition study part. Both DNA extractions and PCR reactions were performed at least in duplicates.

### Adapted Dabney method

The adapted Dabney protocol was provided by Ben Krause-Kyora, Institute of Clinical Molecular Biology (IKMB) Kiel, Germany (personal communication). Digestion and decalcification were performed by incubating different amounts of bone powder overnight (12 –24 h) in 960 µL of 450 mM EDTA pH 8.0 and 40 µL proteinase K (20 mg/mL) at 37 °C and speed 20 in a Tube Revolver Rotator (Thermo Scientific). After incubation, samples were centrifuged at 6,000 rpm (Centrifuge 5415 C, Eppendorf, *r* = 7.3 cm at 2.0 mL tubes) for 2 min and the supernatant of the lysate was transferred to the High Pure Extender Assemblies (High Pure Viral Nucleic Acid Kit, Roche) filled with 10 mL Binding Buffer (Qiagen, Hilden Germany). The High Pure Extender Assemblies were centrifuged at max. 1,500 rpm (Heraeus Multifuge X1R, Thermo Scientific, *r* = 16.5 cm for rotor TX200) at reduced acceleration (acceleration: 5, deceleration: 9) for 6 min. The High Pure filter tubes, which were still connected to the extenders, were removed from the 50 mL Tube and transferred to a collection tube (High Pure Viral Nucleic Acid Kit, Roche), while the High Pure Extenders were irreversibly removed and discarded from the High Pure filter tubes. All filter tubes were centrifuged until dry at 1,400 rpm for 30 s and again at 6,000 rpm for 1 min. The flow-through was discarded. Subsequently, 450 µL of the wash buffer (Roche High Pure Viral Nucleic Acid Kit) was added to the filter tubes and centrifuged at 8,000 rpm for 1 min. The filter tubes were transferred to new collection tubes and the wash step was repeated. Then a dry spin step at 1,400 rpm for 30 s was performed and the filter tubes were placed in 1.5 mL tubes. Finally, the DNA was eluted in two steps with each 30 µL elution buffer (Roche High Pure Viral Nucleic Acid Kit) with an incubation step of 5 min at room temperature and a centrifugation step at 14,000 rpm for 1 min. Extracts were stored until analysis at -20 °C (Supplement Fig. 1 – Workflow Dabney).

#### *Inhibition testing*

For internal validation of the method as a routine application, co-extraction was performed with the two common inhibitors expected in the majority of bone samples, humic acid and calcium (based on a study by Rothe & Nagy [6]). The study was carried out with recent bone material to ensure successful DNA extraction and to be able to evaluate the influence of inhibitors. One metatarsal and one metacarpal bone of an individual with an estimated post-mortem interval (PMI) of seven months were prepared for DNA extraction as described above. The bone powder from the two bone fragments was homogenized to ensure that the same starting material for all experiments was used and to allow for comparability of the results. A total of 13 sample tubes were filled with 100 mg bone powder each and lysed overnight as described. Prior to extraction, 0–5,000 µg of humic acid (Sigma-Aldrich, Germany) or 0–2,050 mg calcium chloride (CaCl_2_) (AppliChem, Germany) were added to the bone samples. A sample without additional inhibitors served as positive control. Working solutions of the inhibitor stock solutions with 25 µg/µL humic acid and 5 M CaCl_2_ were prepared according to Supplement Table 2.

**Table 2:** Statistical evaluation of the DNA quantification results between the Dabney method and bead-based method by using paired Wilcoxon sign-rank test (ɑ = 0.05). *: significant (< 0.05), **: highly significant (< 0.01), ***: very highly significant (< 0.001)

Parameter	*p*-value	Significance
Small fragment	0.0003847	***
Large fragment	0.0009824	***
Degradation index	0.003333	**

#### *Variation of bone powder amount*

In addition, we tested different input quantities of bone powder as part of our method validation. The evaluation of the employed amount of bone powder was based on the study by Xavier et al. [2]. As in the inhibition study, bone powder from recent bone material was used. All extractions were carried out according to the protocol mentioned above. The tested amounts of bone powder were 50 mg, 100 mg (2 × 50 mg), 150 mg, 150 mg (3 × 50 mg), 250 mg and 300 mg (6 × 50 mg). The separately decalcified and lysed 50 mg aliquots were then extracted (two or three of these replicates, respectively) consecutively through the same column. For the extraction of the 300 mg sample, two different spin columns each filled with 150 mg bone powder were used. The DNA was eluted from the silica membrane in two steps with 30 µL elution solution each, or in the case of the 300 mg (2 × 150 mg) replicate, with two times 15 µL per elution step. The eluates from two spin columns were combined to a final volume of 60 µL (Supplement Fig. 2).

**Fig. 2:**
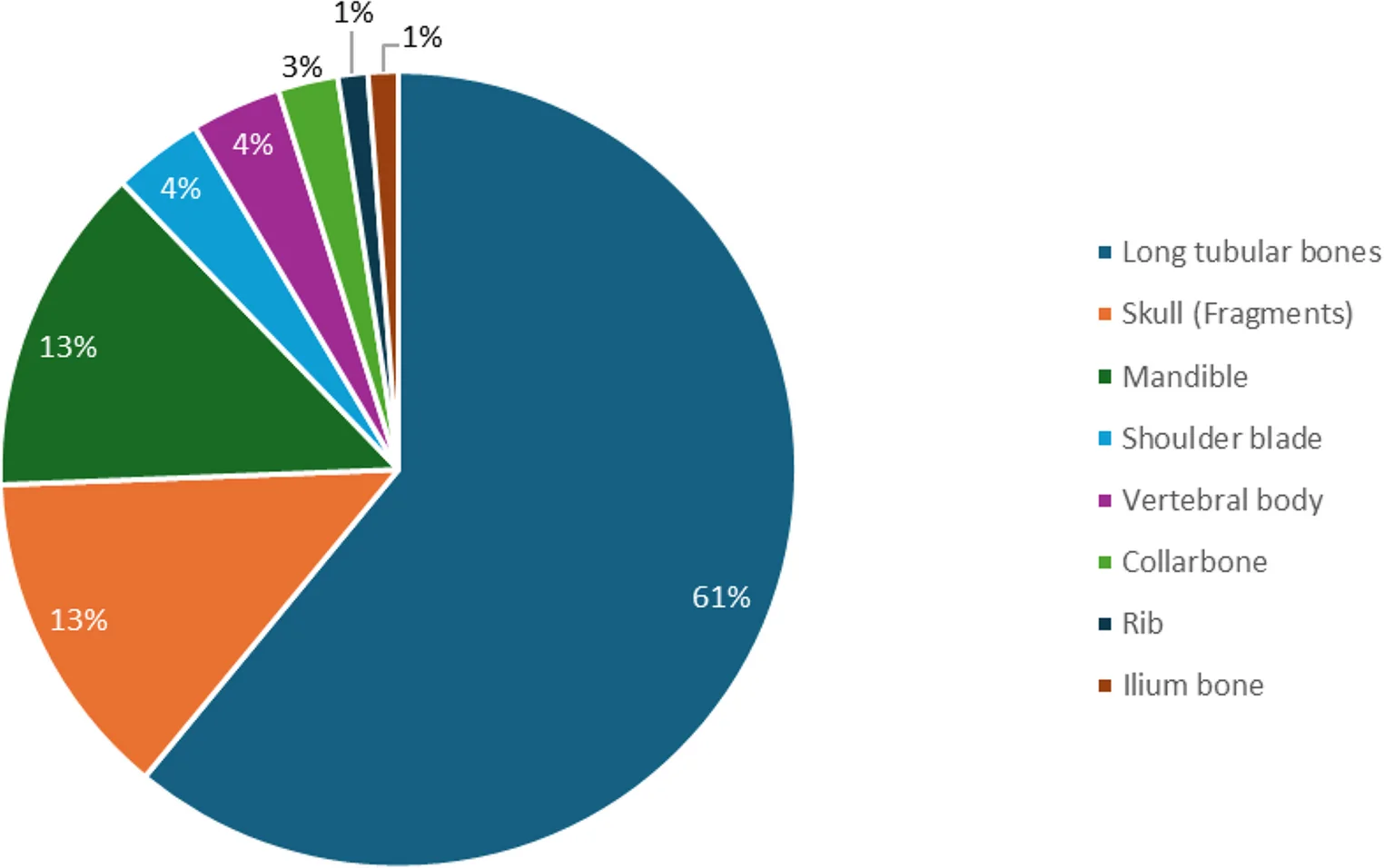
Anatomical distribution of human-specific beach bone finds from the two Institutes of Forensic Medicine in Kiel and Lübeck from 2009 to 2024

### Bead-based method

For the bead-based method, an adapted protocol of the Bone DNA extraction Kit (Promega) was applied. Bone lysis was performed by incubating 100 mg of bone powder in the bone lysis cocktail A containing 355.5 µL of the demineralization buffer, 35.6 µL proteinase K (18 mg/mL) and 8.9 µL 1-Thioglycerol for each sample in a thermo shaker at 56 °C for 2.5 h at 1,000 rpm. After incubation, samples were centrifuged at 13,000 rpm (Centrifuge 5415 C, Eppendorf) for 5 min and the supernatant was transferred to a new 1.5 mL tube. Afterwards, the bone lysis cocktail B from the DNA IQ Casework Pro Kit was prepared by mixing 792 µL lysis buffer and 8 µL 1-Thioglycerol for each sample. The extraction was conducted in the Maxwell MDx according to manufactures instructions. The DNA was eluted in 50 µL elution buffer from the DNA IQ Casework Pro Kit. Extracts were stored until analysis at -20 °C.

### Quantification and STR analysis

DNA concentrations were measured by real-time PCR using the PowerQuant Kit (Promega) on a 7500 Real-Time PCR System (ThermoFisher Scientific) and the PowerQuant Analysis software 1.0.0.0 for evaluation of the results. PCR was set up with an in-house validated reduced volume by mixing 2 µL of the sample with 8 µL of the PowerQuant Mastermix to a total reaction volume of 10 µL. All Dabney samples were run in duplicates. Therefore, mean values of these two samples were calculated and used for comparison with the single quantification value of the bead-based extraction method. Depending on the respective DNA yield, samples were concentrated before STR analysis. For this purpose, 30 µL were centrifuged for 15 min at room temperature in a vacuum concentrator (Thermo Scientific™ Savant™ SpeedVac™ DNA 130). Afterwards, STR amplification of the DNA samples was performed according manufacturer’s instructions with the NGM Detect™ Kit (Applied Biosystems) (NGM) in a PCR Cycler ProFlex (Applied Biosystems). A positive control (PK007, Applied Biosystems) was included in every run to verify PCR functionality. Capillary electrophoresis was performed on a 3500 Genetic Analyzer in 36-cm capillaries filled with polymer POP-4 (Applied Biosystems). Data was collected with ABI Prism Data Collection Software 3.1 and processed with the ABI GeneMapper ID-X v 1.6 (Life Technologies) with a threshold of 50 RFU. Reported allele counts were based on composite profiles obtained from the replicates.

### Statistical analysis

To compare the results of the two extraction methods, normality was checked using the Shapiro-Wilk test and the differences were compared using the paired Wilcoxon sign-rank test for the reanalyzed pairs of the same samples and the Mann-Whitney-U test for the entirety of each method as a group. The level of significance was set to 0.05. Multiple testing was addressed by using the Holm-correction. Differences in DNA concentrations between bones from North and Baltic sea were evaluated by median comparison to account for outliers and only calculated for results based on the Dabney method. The statistical analyses were carried out using R Studio software (version 2023.12.1 402) and GraphPad Prism (version 8.4.3).

## Results

The data was collected retrospectively from the years 2009 to 2024. All marine bone finds that were filed in the archives of the Institutes of Legal Medicine in Kiel and Lübeck were included. During this 15-year period, in total 82 human-specific marine bones were morphologically analyzed. From those, 61% were long tubular bones, followed by skull fragments and mandibles (each 13%) (Fig. 2).

**Fig. 3:**
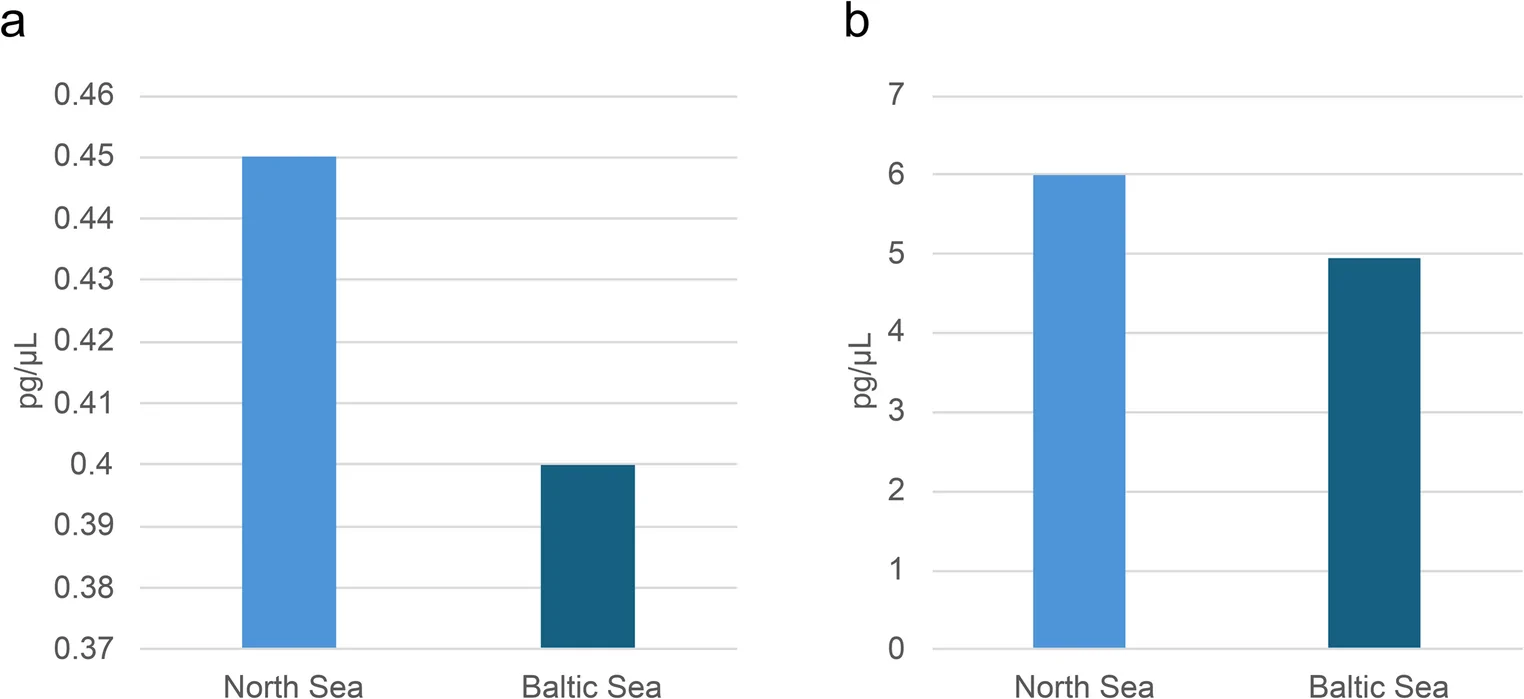
Comparison of median DNA concentrations (in pg/µL) of the small (**a**) and large (**b**) fragment measured by real-time PCR, between the North and Baltic Sea samples extracted with the Dabney method

The bone finds exhibited no differences in the distribution of the type of skeletal element between the North and Baltic sea (Fig. 1). The molecular biological examinations did neither reveal any striking differences in the amount of DNA extracted, nor in the subsequent STR analysis between the North Sea and Baltic Sea finds. To achieve a proper comparison, only the Dabney method results were included in the North Sea vs. Baltic Sea calculations. Due to the small sampling size and statistical outlier (e.g. sample 17, Baltic Sea), we compared the median of the extracted DNA to evaluate the results (Fig. 3).

Some of the beach finds (31 cases) had already been analyzed as part of routine case processing in order to generate DNA profiles for identification purposes. The extractions had been conducted employing the bead-based method (see method section DNA extraction). A total of 18 of these 31 cases could be reanalyzed in this study, as the bones were preserved (Table 1). The extractions were carried out with the adapted Dabney method in duplicates.

### DNA quantification

**Fig. 4:**
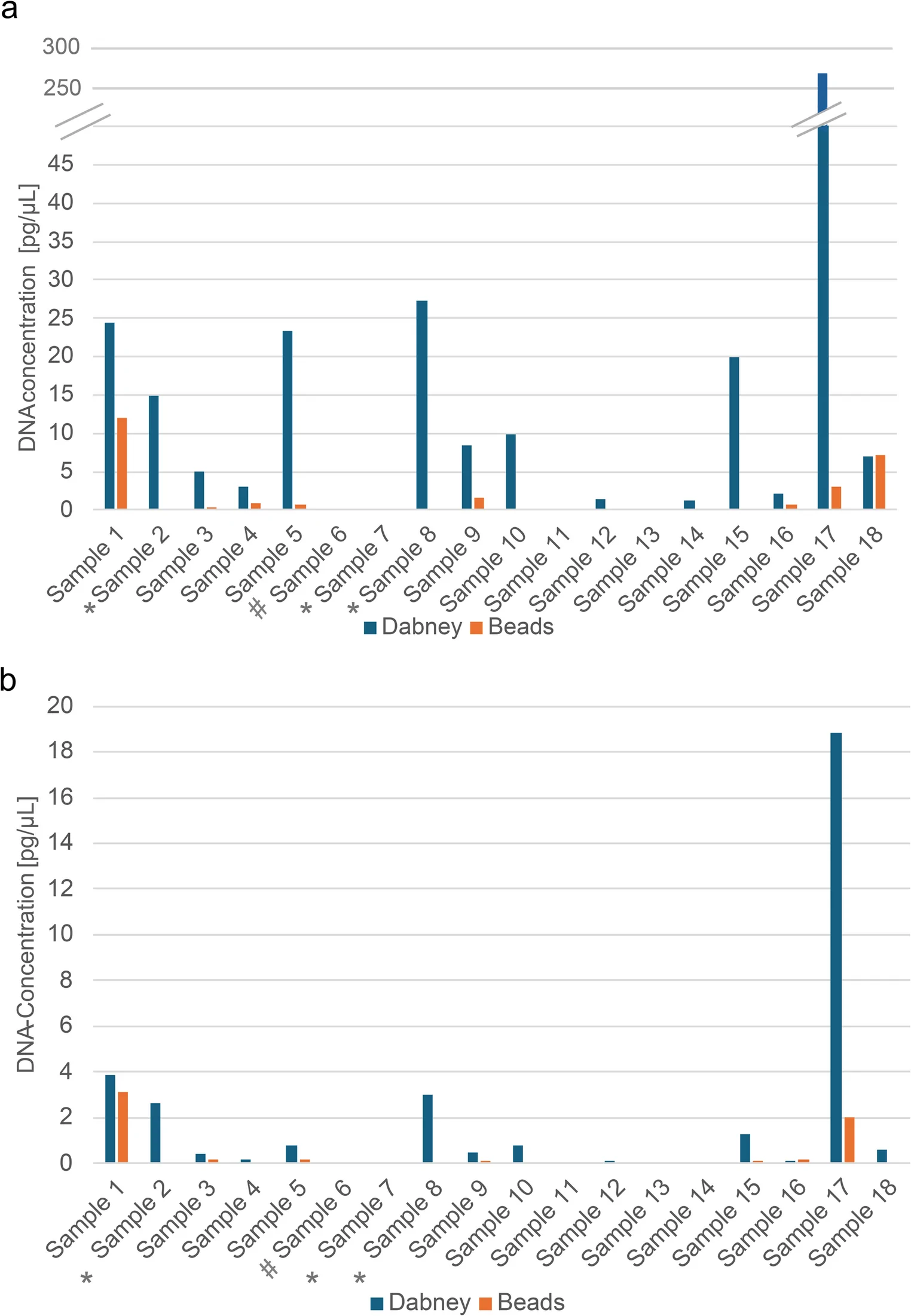
Quantification results of reanalyzed bones for (**a**) small fragment with the length of 84 bp and for (**b**) large fragment with a length of 294 bp. The extracted DNA concentration of the Dabney method (blue) and the bead-based method (orange) were quantified using real-time PCR and displayed in pg/µL. * Bead-based extracted samples without quantification results. # Sample showed contamination

Quantification results are shown in Table 1. There are no DNA quantifications of the bead-based method for samples 2, 7 and 8, because the now utilized quantification method was not yet established at the time these analyses were carried out. The results of sample 6 were also not included in the evaluation due to contamination of the DNA extracts detected in the STR analysis, thereby decreasing the sample size of directly comparable bones to 14. DNA extraction using the Dabney method resulted in significantly higher DNA concentrations for both the small and the large fragments (Table 2), up to 98-fold at sample 10, while the individual differences showed widely variant ratios (Fig. 4a and b). Considered the quantification of both methods as groups instead of pairs, the Dabney method still yields significantly higher DNA concentrations for the small fragments, but not for the long fragments (Supplement Table 3 and Supplement Fig. 3).

### DNA profiling

**Fig. 5:**
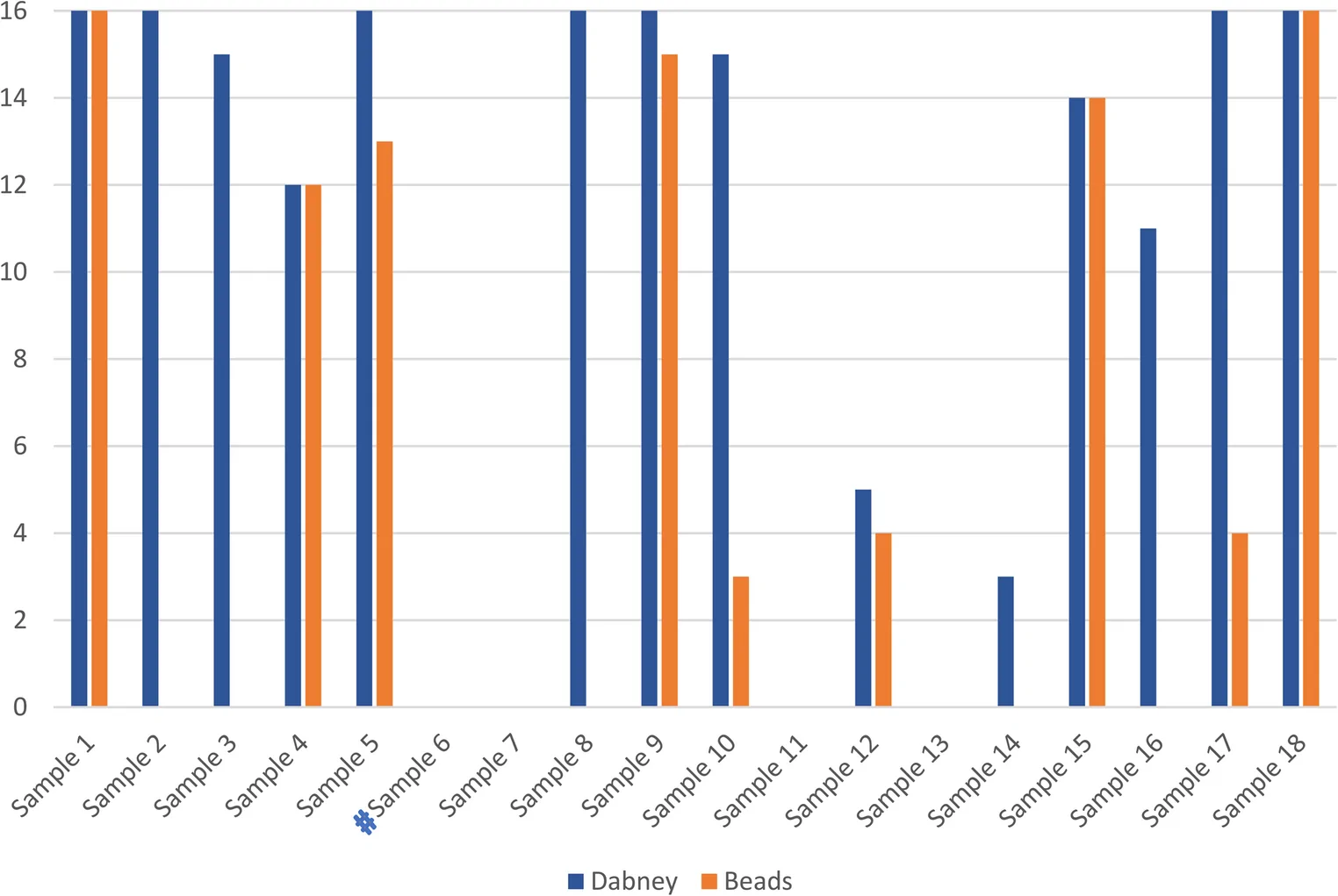
Comparison of the number of called loci detected in the reanalyzed bone samples using the Dabney method (blue) and the bead-based method (orange). A profile was considered complete with 16 Systems. # Sample showed contamination

The Dabney extracted samples led to significantly better STR profiles (Fig. 5, Supplement Fig. 3). The contaminated profile of sample 6 was excluded from evaluation. Compared to two complete profiles (12%) with the bead-based method, seven of the 17 reanalyzed bones resulted in complete profiles (41%). The largest difference between the two analyses was found in sample 2: the Dabney method was able to generate a complete profile, whereas no result at all was obtained with the bead-based method. Furthermore, for samples 10 and 17, the Dabney method resulted in enhanced and nearly complete profiles, while the bead-based method yielded only partial profiles with single alleles. All profiles showed a ski slope-like decrease in signal intensity with the increasing length of the STR amplicons, with preferably allele drop-out in these systems (data not shown).

### Assessment of degradation

**Fig. 6:**
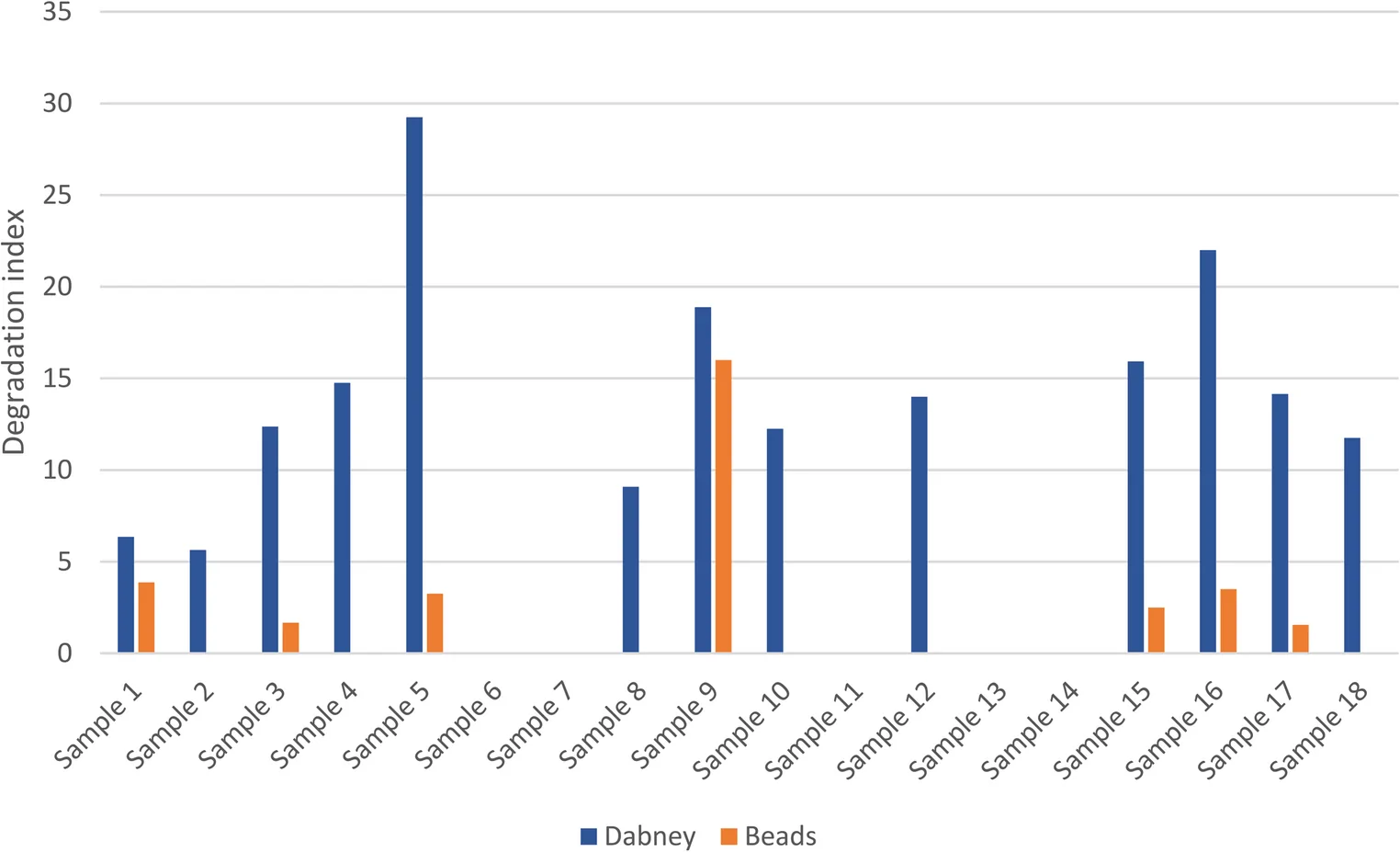
Comparison of the degradation indices detected in the reanalyzed bone samples using the Dabney method (blue) and the bead-based method (orange)

**Fig. 7:**
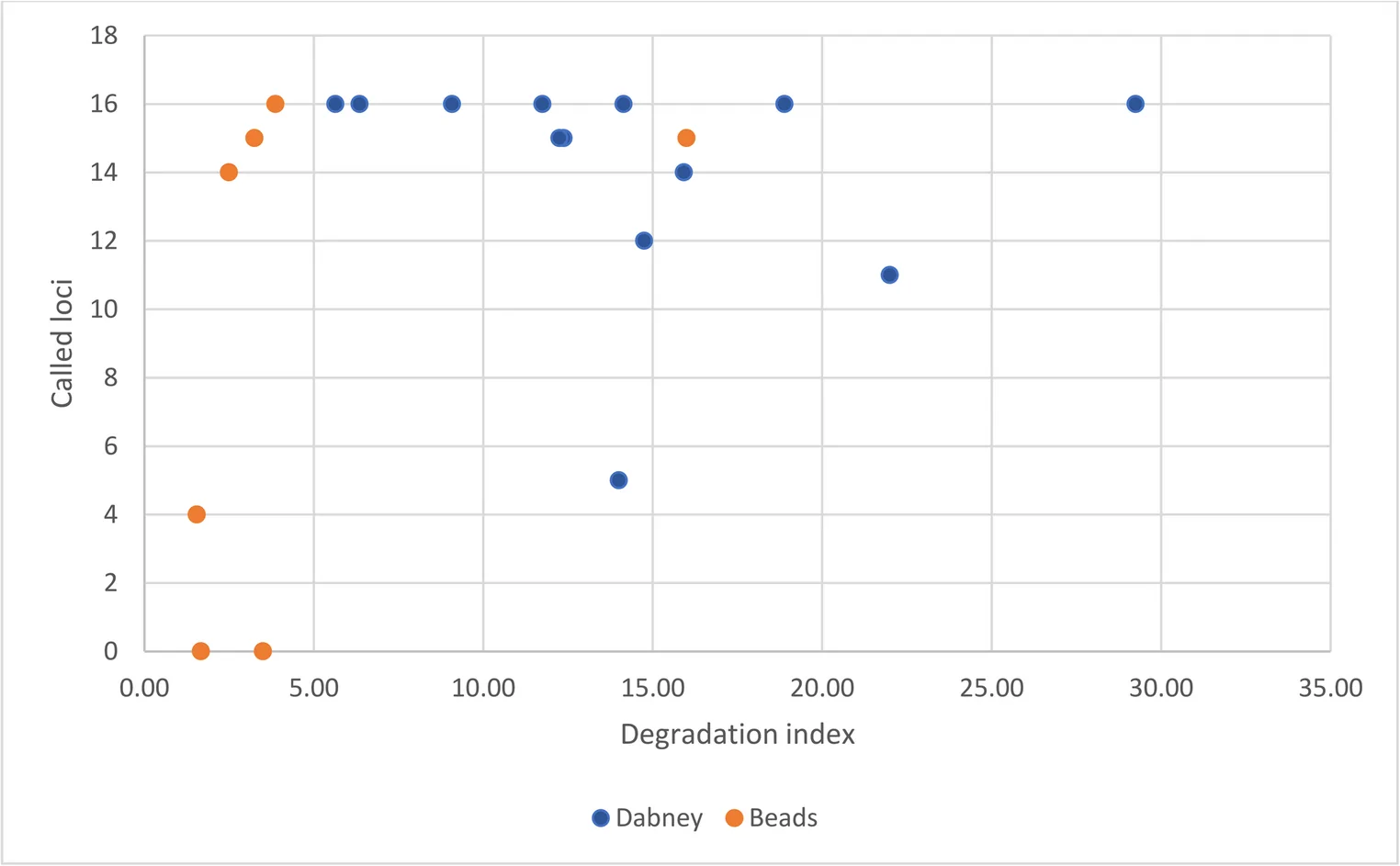
Scatter plot of the degradation indices compared to the number of called loci for the Dabney method (blue) and the bead-based method (orange)

No degradation index (calculated by dividing the concentrations from the short and long fragment) could be determined for the bead-based extracted samples 2, 7 and 8, due to the above-mentioned missing quantification. For the samples 4, 10, 11, 12, 13, 14 and 18 from the bead-based extracts, no DNA was detected in the quantification, therefore no degradation index could be calculated for these samples. The same applies for the Dabney extracted samples 7, 11, 13 and 14. For all the remaining samples extracted using the Dabney method, a degradation index greater than 5 was determined, indicating strongly degraded DNA. In addition, the extracted DNA showed consistently and significantly (Supplement Fig. 3) higher degradation indices than the bead-based extracted DNA (Fig. 6). The relationship between the degradation index and the quality of the correlating STR profiles (number of called loci) is shown in Fig. 7.

### Robustness against Inhibitors

For the calcium test, complete flow of the first four inhibition stages (5–165 mg) could only be achieved after repeated centrifugation steps due to turbidity. Above 555 mg CaCl_2_, the membrane clogged completely. Therefore, only the samples with 5, 50, 100 and 165 mg CaCl_2_ were measured. DNA could only be quantified for the sample with 5 mg CaCl_2_. Compared to the positive control without inhibitor (62.2 pg/µL), the DNA concentration was almost twice as high (118.5 pg/µL). STR profiling using NGM and capillary electrophoresis resulted in a profile with 1 drop-out. No profiles could be generated from any of the other samples tested (Supplement Table 4).

Using humic acid, the extraction was not hindered by any amount used. A decrease of DNA yield was observed with increasing amounts of humic acid, from 112.3 pg/µL using 50 µg humic acid to 4.8 pg/µL using 2,500 µg. With 5,000 µg, the quantification reaction failed. Despite increasing brown coloration of the extracts with increasing amounts of humic acid, the DNA extraction was not mechanically affected and an eluate was obtained for all tested concentration levels. STR profiles could only be generated for samples containing up to 500 µg humic acid, with drop-outs occurring already with 250 µg humic acid input. The internal quality controls (IQCS/IQCL) of the NGM kit in contrast showed no evidence of inhibition. However, the peak heights of the samples decrease in longer fragments (Supplement Table 5).

For the Internal PCR control (IPC) of both tested inhibitors (quantified in the same qPCR run), a non-template extraction control was included. Deviations from the IPC reference value were monitored in addition to the regular IPC shift provided by the PowerQuant system. A value exceeding 0.3 was regarded as indicative of potential PCR inhibition. For the sample with 500 µg humic acid, a slight PCR inhibition was observed. Above an added quantity of 2,500 µg humic acid, the IPC could not be amplified anymore. STR profiling using the NGM kit resulted in a complete profile for the sample with 50 µg humic acid and an almost complete profile for the samples with 250 µg and 500 µg. At the highest amounts of humic acid (2,500 µg and 5,000 µg), it was no longer possible to create a STR profile (Supplement Table 5).

### Bone powder input variations

**Fig. 8:**
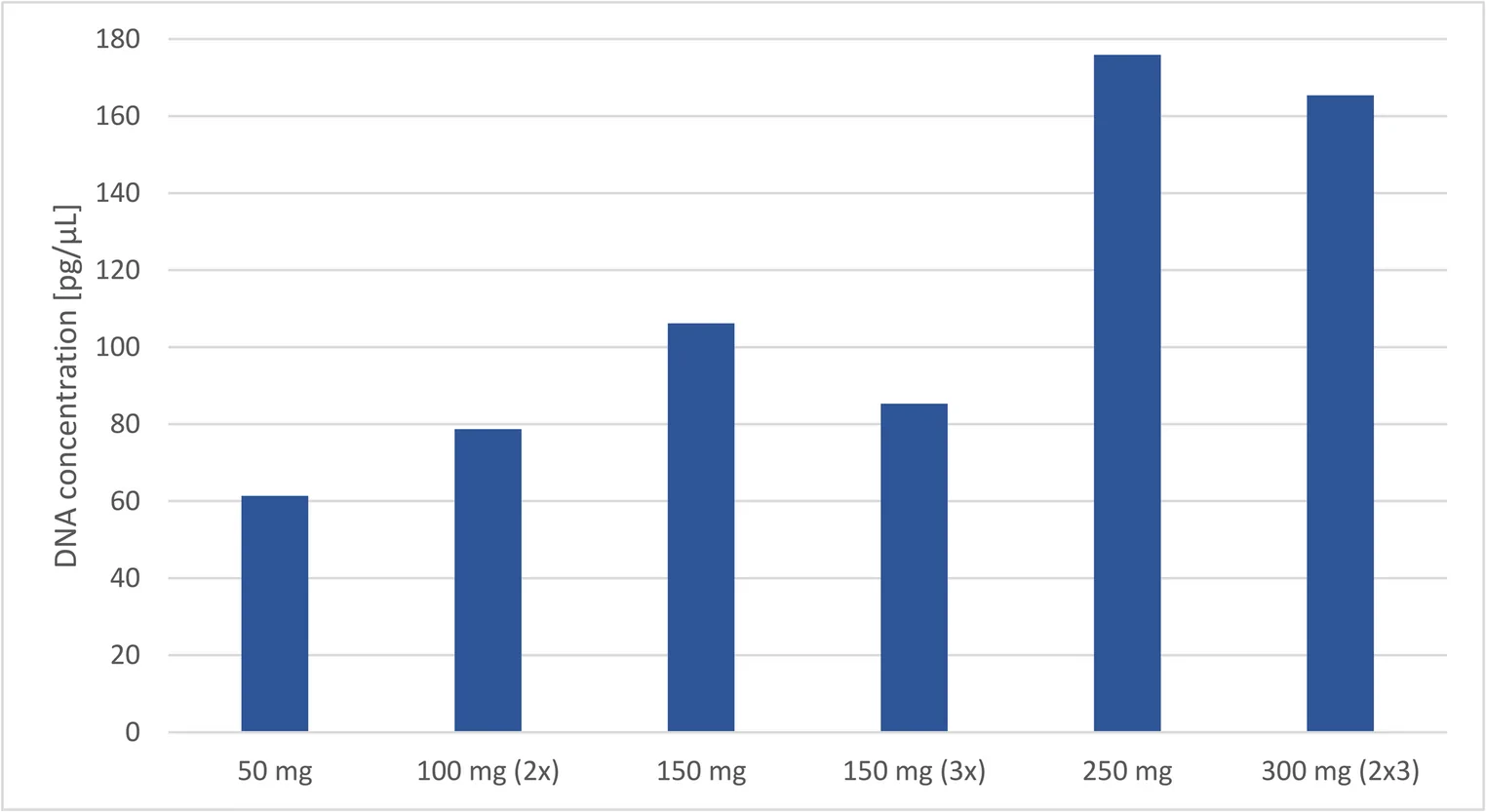
DNA concentration (in pg/µL) as a function of the sample quantity used (in mg). Different sample amounts (50 mg, 100 mg, 150 mg, 250 mg, 300 mg) were used, partly with pooled samples (2 × 50 mg, 3 × 50 mg)

Digestion and decalcification were not influenced by different amounts of bone powder. Further processing of the DNA was comparable to the conventional protocol with 100 mg. The amount of extracted DNA increases with the amount of bone powder used. Noticeably, the pooled sample with 3 × 50 mg resulted in less DNA yield than the respective 150 mg sample (Fig. 8). For the latter sample, a PCR inhibiton with an IPC Cq shift of 1.45 was detected. In comparison, the non-pooled sample resulted in an IPC Cq shift of -0.14. Saturation of the membrane was calculated by multiplying the maximum quantified DNA concentration of 175.8 pg/µL with the total elution volume of 60 µL, to a product yield of approximately 10.55 ng.

## Discussion

The retrospective study of the marine bone finds provides interesting insights into the frequency, anatomical distribution and condition of the bone finds from the North and Baltic Seas. At a total of 82 human-specific finds, 61% were long tubular bones, indicating a certain prevalence for discovery. A recent study from the German state of Hesse provides an overview of bone finds there [7], with long bones also contributing the most (33.7%, 412 of a total of 1,223). One explanation for this observation is probably the bone morphology, which makes them easily recognizable as of potential human origin by (beach) walkers. Furthermore, this type of bone has been demonstrated to be resilient in marine environments, at least over the time span of 14 month [8]. No distinct differences were found in the anatomical distribution of the bone finds between the two seas, however, the differences in the state of preservation were observed. Still, their influence seems neglectable in this sample set.

### Sample selection

As marine bone discoveries are usually individual finds, it is not possible to select the most suitable bone element as recommended by various publications [5, 9, 10]. However, a targeted selection of the sampling site is advisable for maximizing the DNA yield. Depending on the sampled site, different bone parts can vary greatly in their compactness and DNA content [11, 12]. Therefore, selection of the sample location for the reanalysis using Dabney was based on the working instructions published by the International Commission on Missing Persons (ICMP), which was published after the study by Hines et al. [5] and includes a catalogue of the most compact bone areas of the individual bone parts [4].

Although a comparison of the DNA yield between different bone sample sites was not the focus of the present study, we experienced by conducting a direct comparison of two different sample sites from the same skull (sample 2, Supplement Fig. 4) that it is indeed beneficial for DNA recovery to choose the sample site according to published recommendations.

### Reanalyzed sample quality

In comparison to the bead-based method, the Dabney method shows significant improvements in efficiency, particularly in terms of DNA concentration and the quality of the subsequently generated STR profiles. However, all samples showed a degradation index > 5, indicating highly degraded DNA. This can only be explained by the substantially higher number of amplified small fragments, causing an increased degradation index (calculated as the ration of short fragments and large fragments), while still resulting in better profiles (Fig. 7). Compared to the bead-based method, this emphasizes a clear advantage of the Dabney method in terms of efficiency when analyzing heavily degraded samples. These results confirm the findings from previous studies which describe the Dabney method as optimized for the extraction of shorter DNA fragments [1, 2]. Studies by Rothe and Nagy [6] as well as Castella et al. [13], in which bones, saliva and blood were analyzed, also demonstrated that silica membrane methods delivered significantly better results than methods employing silica beads. Additionally, the continuous rotation during overnight lysis, with the bone powder surfaces more evenly presented to the buffer, presumably resulted in improved demineralization, in alignment with other studies highlighting this as an important aspect for better extraction results [14].

### DNA profiling

The results of the STR analysis demonstrate that the DNA extracted by the Dabney method generates profiles of enhanced quality and suitable for forensic (database) comparisons. The STR analysis of sample 2 should be emphasized in particular, as a complete STR profile could be created using the Dabney method, while the bead-based method did not lead to any profiling result. For other samples, such as samples 10 and 17, the Dabney method also led to notably higher numbers of called loci in the DNA profiles (Fig. 5).

Another exemplary gain of knowledge could be obtained in sample 9. In the first analysis using the bead-based method, a presumably nearly complete profile with a large number of homozygous systems was obtained. However, the reanalysis with the Dabney method revealed that 5 systems assumed to be homozygous were actually heterozygous. A noteworthy additional observation was the detection that the skull belonged to a male individual, whereas the original analysis had – incorrectly – indicated a female individual (Supplement Fig. 5).

Still, it shall be noted that we chose the approach of a 50 RFU analytical threshold for allele calling, although we are aware of the risk of erroneously interpreting profiles as either complete or incomplete, due to misinterpretation of homozygosity or by miscalling artefacts.

### Robustness against inhibitors

Focusing on practice oriented outcomes, bone powder was utilized for the inhibition study, in contrast to the study of Rothe and Nagy [6], who worked with purified cells. The use of bone powder renders comparability and standardization more difficult, since the DNA can be unevenly distributed in the samples. This becomes particularly evident in the analyses of the positive control of the inhibition study, which delivered worse results than samples with inhibitors added.

The CaCl_2_ tests indicate that the Dabney method already reaches its limitations at an inhibitor addition of 50 mg. When 5 mg of CaCl_2_ was added, a DNA concentration almost twice as high as that of the positive control was detected. Whether this phenomenon was caused by the CaCl_2_ or by the bone powder used for this sample remains uncertain. However, a similar result was observed by Rothe and Nagy [6]. One explanation could be, as presented by Chen et al. [15], that high salt concentrations favor an increase of hydrogen bonds and hydrophobic interactions, while at the same time reducing the electrostatic repulsive forces between DNA and silica surface, resulting in a stronger adsorption. No STR analysis could be performed for any samples tested with CaCl_2_ input amounts above 5 mg.

In contrast, the humic acid tests show a gradual decrease in DNA yield with increasing inhibitor concentration. STR profiles could be, albeit with drop-outs, be created with an input amount 500 µg, but no longer at 2,500 µg humic acid, which corresponds to data from both PowerQuant and NGM Detect manuals by the manufacturers.

The results of the present study are comparable with the findings from Rothe and Nagy [6], who concluded that the efficiency of DNA extraction depends heavily on the method used and the specific conditions of the sample. Still, the Dabney method proves to be a robust DNA extraction method with satisfactory results under moderate inhibitor concentrations, but with limitations at higher concentrations of CaCl_2_ and humic acid.

### Variation of the amount of bone powder

**Fig. 9:**
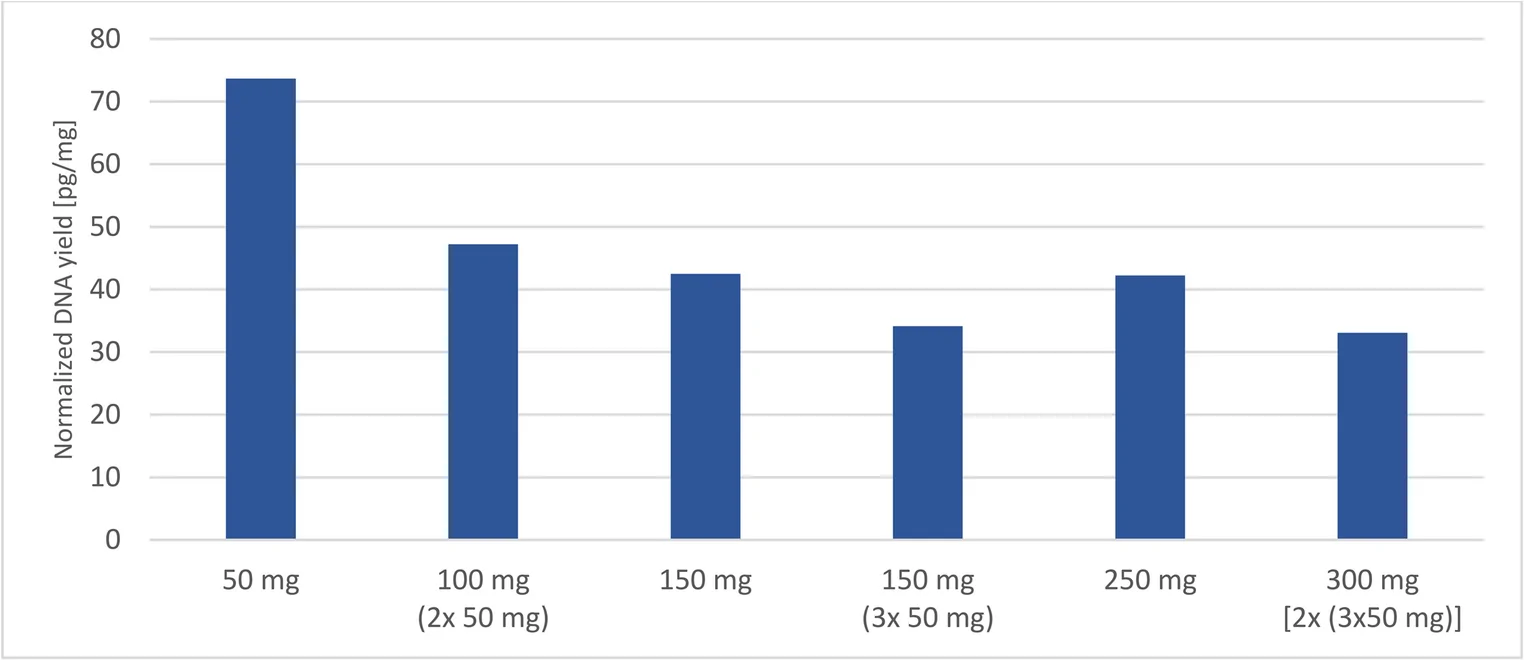
Normalized DNA concentration (in pg/mg). Different sample amounts (50 mg, 100 mg, 150 mg, 250 mg, 300 mg) were used, partly with pooled samples (2 × 50 mg, 3 × 50 mg)

A positive correlation between the increasing amount of bone powder and the total DNA yield was observed, although the normalized DNA amount per mg bone powder decreased (Fig. 9). Accordingly, Xavier et al. [2] observed a lower normalized DNA yield per mg bone powder when doubling the input to 100 mg in the same extraction. In comparison, the pooled sample of 3 × 50 mg bone powder in our study led to a noticeably lower DNA concentration as well as a lower normalized DNA yield per mg bone powder than the non-pooled 150 mg sample, which is contradictory to published data [2, 16]. However, as the experiment was only performed once, it is not advisable to draw any firm conclusions. The lower amount of DNA measured in the pooled sample in our study could also be explained by the simultaneously increased extraction of inhibitors. The 150 mg sample pooled from 3 × 50 mg showed a substantially higher amount of PCR inhibitors with an IPC Cq shift of 1.45 compared to -0.14 for the non-pooled sample. A similar result is also shown by Rothe and Nagy [6], who found critical amounts of inhibitors after pooling samples on a silica membrane. Although the experiment was conducted only once without replicates, the results indicate that a saturation of the silica membrane was reached in our cases with an input of about 250 mg bone powder (Fig. 8), which corresponds to approx. 10.55 ng DNA. This illustrates that adapting the amount of bone powder, especially in samples with limited DNA concentration (e.g. in aged or degraded bones), serves as an efficient attempt to enhance DNA yield.

### Limitations

As this study was conducted on real bone finds, it lacks the controlled comparability of a lab study done with bones from body donors. Timeframes of water exposure and environmental conditions experienced by the bones are eventually unknown. Furthermore, we are aware that the sample size is limited and a systematic comparison of different sample sites was not the focus of our study; their change rather an inclusive part of the extraction improvement protocol. These factors, along with other possible inhibitors which bones could be exposed to in other marine conditions across the world could not only confirm the strength of the presented method but also provide insight to its limitations up to a point where a different workflow might be more advisable.

## Conclusion

The reanalyzes of the marine bones demonstrated that the DNA extraction method according to Dabney offers great potential for forensic DNA analysis to successfully process recent and aged, sea water exposed or severely damaged bone material for identification purposes. The obtained results also emphasize the importance of diligent sample preparation, as the uneven distribution of DNA in bone samples remains challenging, yet are maximizable by a careful selection of advised sampling sites. An increase of bone powder until membrane saturation was proven to be possible and is advised for low template samples. Yet, as only a limited regional sample set could be used, further validation studies with bone material from equal or different environmental conditions and employing alternative inhibitors should be conducted.

## Supplementary Information

Below is the link to the electronic supplementary material.

Supplementary Material 1 (1.79 MB)

## Data Availability

All data generated or analyzed during this study are included in this published article [and its supplementary information files].
