# Supplementary material for: Improved DNA extraction from marine bones: A 15-year retrospective evaluation using an adapted Dabney extraction method
Source: Int J Legal Med. 2026 Jul 17;140(6):3849–60. doi: 10.1007/s00414-026-03923-w (PMC13658333; doi:10.1007/s00414-026-03923-w)
Supplement: Supplementary file 1 [file 414_2026_3923_MOESM1_ESM.docx]

**Supplement Table 1.** Reanalyzed bone finds and discovery sites.

| Sample | Bones | Location | Ocean |
| --- | --- | --- | --- |
| 1 | Femur | Sylt | North Sea |
| 2 | Cranium | Fehmarnsund | Baltic Sea |
| 3 | Tibia | Pelzerhaken | Baltic Sea |
| 4 | Humerus | Schleuseninsel | Baltic Sea |
| 5 | Femur | Wittdün (Amrum) | North Sea |
| 6 | Femur | Sylt | North Sea |
| 7 | Scapula | Wittdün (Amrum) | North Sea |
| 8 | Mandibula | Westerhever | North Sea |
| 9 | Part of a Cranium | Eckernförde | Baltic Sea |
| 10 | Femur | Wittdün (Amrum) | North Sea |
| 11 | Presumably Femur | Utersum (Föhr) | North Sea |
| 12 | Tibia | St. Peter-Ording | North Sea |
| 13 | Femur | Eckernförde | Baltic Sea |
| 14 | Scapula | St. Peter Ording | North Sea |
| 15 | Femur | Westerhever | North Sea |
| 16 | Femur | Westerhever | North Sea |
| 17 | Tibia | Pelzerhaken | Baltic Sea |
| 18 | Femur | Hasselberg | Baltic Sea |

**Supplement Table 2.** Tested calcium chloride and humic acid concentrations.

| Calcium chloride (5M) | | Humic acid (25µg/µL) | |
| --- | --- | --- | --- |
|  |  |  |  |
| Stock solution: 27.75 g in 50 ml water | | Stock solution: 25 mg in 1000 µL water | |
|  | |  | |
| Amount of 5M CaCl_2_ in the extraction | **Volume from the stock solution** | **Amount of Humic acid (25 µg/µL) in the extraction** | **Volume from the stock solution** |
| 0 | 0 | 0 | 0 |
| 5 mg | 9 µL | 50 µg | 2 µL |
| 50 mg | 90 µL | 250 µg | 10 µL |
| 100 mg | 180 µL | 500 µg | 20 µL |
| 165 mg | 300 µL | 2500 µg | 100 µL |
| 555 mg | 1000 µL | 5000 µg | 200 µL |
| 1110 mg | 2000 µL |  |  |
| 2050 mg | 3700 µL |  |  |

**Supplement Table 3.** Statistical evaluation of the DNA quantification results between the Dabney method and bead-based method for the whole groups by using Mann-Whitney-U test (ɑ = 0.05). n.s.: not significant, *: significant (< 0.05), **: highly significant (< 0.01).

| Parameter | p-value | Significance |
| --- | --- | --- |
| Small fragment | 0.0126 | * |
| Large fragment | 0.08342 | n.s. |
| Degradation index | 0.001345 | ** |

**Supplement Table 4.** Comparison of DNA extraction with different calcium chloride amounts. IPC Cq: Internal PCR control quantification cycle (Real-Time-PCR), IQC: Internal Quality Control (NGM-Kit), n.a.: not analysed, because the membrane was blocked; n.a.p.: no analysis possible; /: no quantification result.

| Amount of CaCl_2_ (mg) | Average peak area | Drop-outs | DNA concentration (pg/µL) | IPC Cq | IQC |
| --- | --- | --- | --- | --- | --- |
| No | 7846 | 2 | 62.2 | 20.33 | 0.69 |
| 5 | 7993 | 1 | 118.5 | 20.53 | 1.04 |
| 50 | n.a.p. | n.a.p. | / | / | n.a.p. |
| 100 | n.a.p. | n.a.p. | / | / | n.a.p. |
| 165 | n.a.p. | n.a.p. | / | / | n.a.p. |
| 555 | n. a. | n. a. | n. a. | n. a. | n. a. |
| 1,110 | n. a. | n. a. | n. a. | n. a. | n. a. |
| 2,050 | n. a. | n. a. | n. a. | n. a. | n. a. |

**Supplement Table 5.** Comparison of DNA extraction with different humic acid amounts. IPC Cq: Internal PCR control quantification cycle (Real-Time-PCR), IQC: Internal Quality Control (NGM-Kit), n.a.: not analysed, because the membrane was blocked; n.a.p.: no analysis possible due to strong inhibition; /: no quantification result.

| Amount of humic acid (µg) | Average peak area | Drop-outs | DNA concentration (pg/µL) | IPC Cq | IQC |
| --- | --- | --- | --- | --- | --- |
| No | 7846 | 2 | 62.2 | 20.33 | 0.69 |
| 50 | 12904 | 0 | 112.3 | 20.67 | 0.63 |
| 250 | 9352 | 2 | 88.1 | 20.44 | 0.75 |
| 500 | 9104 | 2 | 75.2 | 21.00 | 0.75 |
| 2,500 | n.a.p. | n.a.p. | 4.8 | / | n.a.p. |
| 5,000 | n.a.p. | n.a.p. | / | / | n.a.p. |

**Supplement Fig. 1.** Workflow of the adapted Dabney extraction method.

**Supplement Fig. 2.** Workflow of combined samples for bone powder quantities 100 mg, 150 mg and 300 mg.

**Supplement Fig. 3.** Mean values and their standard deviation of the Dabney method (blue) and the bead-based method (orange) for the quantification parameters (a) small and (b) large fragment, (c) the degradation index and for (d) the called loci. Significance tested by Mann-Whitney-U test using R. n.s.: not significant, *: significant (< 0.05), **: highly significant (< 0.01).

**Supplement Fig. 4.** Comparison of the sampled sites and the ICMP work instruction for sample 2. First sample site (mean 0.2 pg/µl), second sample site (mean 14.95 pg/µl).

**Supplement Fig. 5.** Illustration of the electropherograms of sample 9, which were generated using the NGM kit. The profile from the Dabney method (a) is shown on the left and that from the bead-based extraction method (b) on the right. The loci in which heterozygosity was detected in the reanalysis were circled instead of a falsely assumed homozygosity. The peaks represent the amplified alleles, with the x-axis indicating the fragment size in base pairs and the y-axis the signal intensity (RFU). The numerical values below the peaks indicate the corresponding signal strengths.

**Extraction comparison of recent bones**

As part of the pilot study, recent bone finds from a grave with an estimated burial time of two years and bones from a recycling center with no further information on burial time were also examined. The samples from the grave comprised two teeth and a femur sample, while the sample from the recycling center was obtained from a skull. The analysis of the recent bone samples yielded almost complete DNA profiles with both extraction methods (Supplement Fig. 6). Furthermore, the quantification results of the two different DNA fragments show that the Dabney method also resulted in more smaller fragments from recent bones (Supplement Fig. 7). These results illustrate that the Dabney method is an efficient extraction method for both recent and heavily degraded samples, such as marine bones.

**Supplement Fig. 6.** Called loci of recent bone finds for the Dabney method (blue) and the bead-based method (orange). The profile was considered complete with 16 systems.

|  | Dabney | Beads |
| --- | --- | --- |
| Femur Skeleton | 16 | 16 |
| Teeth Skeleton | 16 | 16 |
| Teeth Skeleton | 16 | 16 |
| Cranium Recycling center | 16 | 15 |
| Cranium Recycling center | 16 | 15 |

a

b

DNA concentration [pg/µL]

|  | small fragment Dabney | small fragment Beads |
| --- | --- | --- |
| Femur Skeleton | 6.1 | 2 |
| Teeth Skeleton | 22.1 | 6.3 |
| Teeth Skeleton | 5 | 0.6 |
| Cranium Recycling center | 27.2 | 9.5 |
| Cranium Recycling center | 21.4 | 8.4 |

DNA concentration [pg/µL]

|  | large fragment Dabney | large fragment Beads |
| --- | --- | --- |
| Femur Skeleton | 2 | 0.4 |
| Teeth Skeleton | 5 | 2 |
| Teeth Skeleton | 0.8 | 0.1 |
| Cranium Recycling center | 0.7 | 0.3 |
| Cranium Recycling center | 0.6 | 0.3 |

**Supplement Fig. 7.** Quantification results of the reanalyzed recent bones. For (a) the small fragment with a length of 84 bp and for (b) the large fragment with a length of 294 bp. The extracted DNA concentration of the Dabney method (blue) and the bead-based method (orange) were quantified using real-time-PCR and displayed in pg/µL.
